# Vacuum-Deposited Cesium Tin Iodide Thin Films with Tunable Thermoelectric Properties

**DOI:** 10.1021/acsaem.2c01936

**Published:** 2022-07-26

**Authors:** Paz Sebastia-Luna, Unnati Pokharel, Bas A. H. Huisman, L. Jan Anton Koster, Francisco Palazon, Henk J. Bolink

**Affiliations:** † Instituto de Ciencia Molecular, ICMol, Universidad de Valencia, 46980 Paterna, Spain; ‡ Zernike Institute for Advanced Materials, University of Groningen, 9747 AG Groningen, The Netherlands; § Departamento de Ingeniería Química y Ambiental, Universidad Politécnica de Cartagena, 30202 Cartagena, Spain

**Keywords:** thermoelectrics, perovskite, tin, thin film, conductivity, Seebeck, room temperature

## Abstract

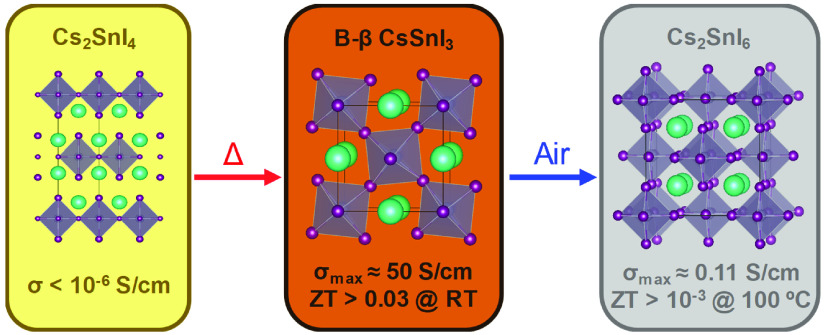

Most current thermoelectric materials have important drawbacks, such as toxicity, scarceness, and peak operating temperatures above 300 °C. Herein, we report the thermoelectric properties of different crystalline phases of Sn-based perovskite thin films. The 2D phase, Cs_2_SnI_4_, is obtained through vacuum thermal deposition and easily converted into the black β phase of CsSnI_3_ (B-β CsSnI_3_) by annealing at 150 °C. B-β CsSnI_3_ is a p-type semiconductor with a figure of merit (ZT) ranging from 0.021 to 0.033 for temperatures below 100 °C, which makes it a promising candidate to power small electronic devices such as wearable sensors which may be interconnected in the so-called Internet of Things. The B-β phase is stable in nitrogen, whereas it spontaneously oxidizes to Cs_2_SnI_6_ upon exposure to air. Cs_2_SnI_6_ shows a negative Seebeck coefficient and an ultralow thermal conductivity. However, the ZT values are 1 order of magnitude lower than for B-β CsSnI_3_ due to a considerably lower electrical conductivity.

## Introduction

Thermoelectric generators (TEGs) represent a very promising source of renewable energy, as they directly convert (waste) heat into electricity.^1,2^ Thermoelectric materials are typically characterized by the figure of merit (ZT)
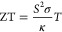
1which depends on the following key parameters: *S* is the Seebeck coefficient, σ is the electrical conductivity, κ is the thermal conductivity, and *T* is the absolute temperature. Thus, to maximize the ZT value of a given material, a large Seebeck coefficient, a high electrical conductivity, and a low thermal conductivity are required.^2,3^ Among the most widely used thermoelectric materials, we find Bi_2_Te_3_, Sb_2_Te_3_, and SnSe, with the latter exhibiting a ZT of 3.1 (at 798 K), the highest reported value so far.^2,4,5^ Nevertheless, the scarceness, toxicity, and high fabrication costs of these materials, especially for tellurides, represent a huge hindrance in their further development.^6,7^ Besides, most of them are used in a single crystal state,^2,4^ which limits their integration in different device architectures. Another major bottleneck comes from the temperature where their operational peak is reached, usually above 300 °C.^8^ It is paramount to decrease this minimum temperature required to widen the possible applications of TEGs and make them a suitable power source for the Internet of Things (IoT) and its many derivatives in industry, agriculture, and wearable healthcare devices.^9^ Because the majority of these applications require room or moderate temperatures, TE materials with good performance below 100 °C are needed.^10^

Organic semiconductors such as poly(3,4-ethylenedioxythiophene) (PEDOT) or doped fullerene derivatives with oligoethylene–glycol (OEG) side chains have been proposed for near room temperature TE.^1,11^ While these are undoubtedly interesting alternatives, the use of such organic semiconductors presents additional challenges: given the large size of the organic dopants (necessary to enhance electrical conductivity and hence ZT), their incorporation into the host material without disrupting the packing and creating additional energetic disorder is challenging.^11^ Furthermore, polymers are unsuitable for high-purity thin film deposition methods based on vacuum sublimation. Hence, inorganic TE materials operating near room temperature are sought after. Currently, some inorganic materials, such Ag_2_Se or Cu_2_Se, have shown good prospects for room temperature applications, but a fine-tuning of the stoichiometry and deposition conditions is needed to achieve the best efficiencies, a process that can be costly and time-consuming.^12,13^ In the search for alternative materials that are easy to process, metal halide perovskites, such as CH_3_NH_3_PbI_3_, FASnI_3_, or CsSnI_3_, have emerged in recent years as potential thermoelectric materials. Indeed, ultralow thermal conductivity and acceptable Seebeck coefficients have been demonstrated.^14−16^ Sn-based halide perovskites surpass their Pb counterparts in thermoelectric performance because of the self-oxidation of Sn^2+^ to Sn^4+^, acting as a self-doping mechanism that enhances their electrical conductivity.^16^ Besides, their toxicity is reduced due to the absence of Pb in their composition. In this work, we focus herein on CsSnI_3_, which furthermore does contain Cs instead of an organic cation, offering much higher thermal stability than organic–inorganic perovskites.^17^ It must be noted that Cs is up to 3 orders of magnitude more abundant on the earth’s crust than Bi or Te, which are common elements for current thermoelectrics.^10^ In fact, Cs is one of the most “underproduced” elements with scope for increased production.^18^ With regards to toxicity, cesium has 40 known isotopes, of which radioactive ^137^Cs is the most toxic and dangerous. In this work, we use its most stable isotope, ^133^Cs, with a much lower toxicity.^19^ Therefore, CsSnI_3_ emerges as a promising alternative to most current thermoelectric compounds such as PbTe, Bi_2_Te_3_, or Sb_2_Te_3_ with regards to element abundance and toxicity.

CsSnI_3_ is known to have four different polymorphs, two of them existing at room temperature (Table 1):^20,21^ a yellow phase, Y CsSnI_3_, with an orthorhombic one-dimensional double-chain structure and an orthorhombic three-dimensional perovskite that is black in color, B-γ CsSnI_3_. On the basis of the literature, annealing Y CsSnI_3_ to 150 °C under an inert atmosphere yields a black cubic perovskite (B-α), which upon cooling back below 150 °C transforms into a black tetragonal phase (B-β) and into the black orthorhombic phase B-γ when it is cooled below 80 °C. Exposing B-γ CsSnI_3_ to air for a short period of time triggers the transformation into Y CsSnI_3_, as this is the species that is thermodynamically more stable at ambient conditions.^20,21^ Eventually, upon prolonged exposure to air, Y CsSnI_3_ evolves to Cs_2_SnI_6_, a vacancy-ordered double perovskite that contains oxidized Sn^4+^ rather than Sn^2+^ ions.^22^ Previous reports have studied the thermoelectric properties of CsSnI_3_ thin films. Saini et al. prepared films grown by the solution process in DMSO:DMF mixtures and using toluene as antisolvent, followed by thermal annealing reaching a ZT of 0.137 at room temperature.^23^ Kontos et al. studied the effect of SnF_2_ doping onto spin-coated CsSnI_3_ films, revealing a change in the electrical resistance upon the exposure to air of the samples.^21^ In the same line, Liu et al. reached a ZT around 0.14 in SnCl_2_-doped CsSnI_3_ films deposited by a sequential evaporation method.^24^ Nevertheless, this performance is only achieved upon the introduction of additional SnCl_2_ into the structure and after exposure of the films to air and humidity for 6 min, while a longer exposure time was found to diminish the ZT again.^24^ Kanatzidis and co-workers studied the thermal and transport properties of a series of CsSnBr_3–*x*_I_*x*_ perovskites, obtaining a ZT for CsSnI_3_ of 0.025 at 300 K that reaches a maximum of 0.15 at 550 K. These results were achieved with bulk (6 × 6 × 1.5 mm) crystals sintered at 923 K for periods of more than 24 h.^25^ In summary, these results demonstrate the potential of CsSnI_3_ thin films for TE. However, the aforementioned protocols include complex solvent processing, need of external dopants or additives, and high-temperature synthesis and/or are very sensitive to air and humidity. More robust and simpler routes are therefore sought after.

**Table 1:**
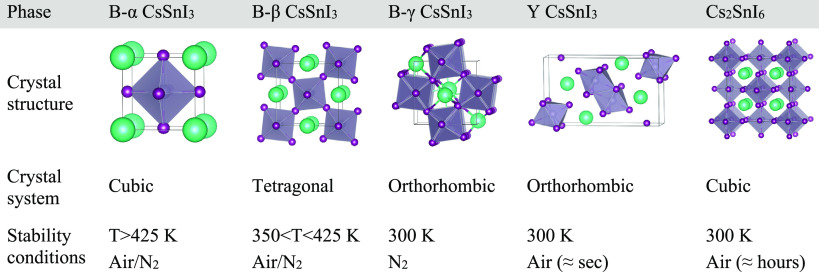
Existing Different Phases and Structures of the Cs–Sn–I System, Their Crystal Structures, and Ambient Conditions for Each Phase Transition^a^

a Transition temperatures are taken from the literature.^20,21^

Here, we focus our research on the study of vacuum deposition of CsSnI_3_ thin films via single source thermal evaporation of presynthesized dry powders. Thermal vacuum deposition shows better thickness control, higher throughput, and higher reproducibility compared to solution processing.^26,27^ Remarkably, the as-synthesized film presents the two-dimensional structure characteristic of Cs_2_SnI_4_, a species reported only theoretically so far. Its electrical conductivity detected is very low but can be increased by several orders of magnitude upon annealing and consequent conversion to B-β CsSnI_3_, achieving a maximum ZT of 0.033.

## Results and Discussion

Following the procedure described in the Experimental Methods, synthesis of the CsSnI_3_ was performed via dry mechanochemical synthesis by ball-milling under nitrogen, thus preventing oxidation and degradation of Sn(II) in contact with the atmosphere. The synthesized CsSnI_3_ is a black powder with a complex X-ray diffraction pattern (see Figure S1). When this diffraction pattern is compared with the known patterns for the yellow phase (Y CsSnI_3_, Inorganic Crystal Structure Database code 262927) and the black gamma phase (B-γ CsSnI_3_, ICSD code 262926), it can be deduced that our as-synthesized powder is a mixture of these two phases.

**Figure 1:**
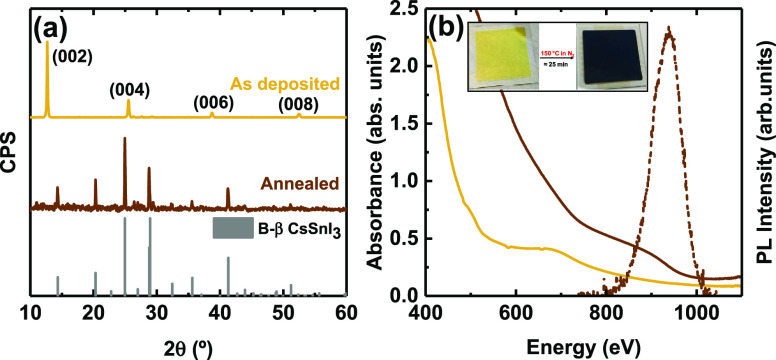
(a) XRD diffractograms and (b) absorbance (solid lines) and photoluminescence (dashed line) spectra of SSVD thin films as-deposited and annealed for 25 min at 150 °C. Panel a shows the ICSD pattern for the black beta phase of CsSnI_3_ (code 262925) and the predicted main crystalline structure for both films. Predicted XRD planes of as-deposited thin films were obtained from the fit presented in Figure S2.

Once the powders were formed, thin films with thicknesses of few hundred nanometers (see the Experimental Methods for more details) were deposited via single-source vacuum deposition (SSVD) inside a high-vacuum chamber. SSVD has been previously shown by us and others to be a fast and reproducible method for depositing pure, stoichiometric materials.^28−30^ In our case, the SSVD of the ball-milled CsSnI_3_ did not lead directly to the deposition of any of the previously mentioned CsSnI_3_ phases, as the XRD diffractogram does not match with any of them (Figure 1a). On the contrary, the few and equally spaced peaks present are reminiscent of a 2D material. Indeed the main diffractogram signals can be well matched with a 2D phase isostructural to Cs_2_Pb(I_0.5_Cl_0.5_)_4_ (Figure S2).^31^ Thus, it is reasonable to ascribe this signal to the crystallization of the 2D phase Cs_2_SnI_4_ (Figure S2), whose main diffraction peaks correspond to the (00*l*) planes. To the best of our knowledge, this phase has only been theoretically reported because of its high instability.^32,33^ Indeed, we attempted the mechanochemical synthesis of Cs_2_SnI_4_, but a mixture of Y CsSnI_3_ and B-γ CsSnI_3_ was formed instead (Figure S3). The formation route of Cs_2_SnI_4_ upon the sublimation of CsSnI_3_ powder still remains unclear to us. We hypothesize that even if the 2D phase is unstable, the high energies supplied by the evaporation process allowed its formation. However, we cannot rule out the coexistence of Cs_2_SnI_4_ in the thin films together with CsSnI_3_ phases. Indeed, minor peaks around 2θ = 27.6° and 2θ = 29.2° do not match the 2D structure and suggest traces of Y CsSnI_3_ and B-γ CsSnI_3_ (Figure S2). The preferential deposition of Cs_2_SnI_4_ took place in a reproducible manner at several evaporation batches of ball-milled CsSnI_3_ (Figure S4), with slight differences in the presence of side phases that could be linked to variations in the air exposure. Elemental analysis by energy dispersive X-ray spectroscopy (EDX) shows a molar ratio of Cs:Sn:I 1:1.5:3.1, very close to the stoichiometry expected for a CsSnI_3_-derived phase (1:1:3) with an excess of Sn ascribed to indium tin oxide (ITO) substrate. The absorption spectrum (Figure 1b) confirms the presence of at least two different species with different absorption intensities. The Tauc plot derived from the absorption spectrum reveals two bandgaps of 2.45 and 1.42 eV which are ascribed to Y CsSnI_3_ (indirect transition) and Cs_2_SnI_4_ (direct transition) phases, respectively, very similar to the literature references (Figure S5).^32,34^

A preliminary study of the electrical properties of the 2D phase revealed a very low conductivity (Figure S6). Such a low conductivity is detrimental for thermoelectric use, as it would lead to a very low ZT value. However, it was possible to convert the poorly conductive Cs_2_SnI_4_ into the more conductive black beta phase of CsSnI_3_ (B-β CsSnI_3_) by thermal annealing at 150 °C under an inert atmosphere (Figure 1a). Figure S7 shows the XRD patterns resulting from *in situ* annealing of Cs_2_SnI_4_ thin films up to 150 °C. Cs_2_SnI_4_ undergoes a gradual conversion to CsSnI_3_, for which 25 min of annealing is required to form a pure black beta phase of CsSnI_3_. This transformation is further evidenced by the increase in optical absorption of the thin film in the 500–950 nm region (Figure 1b), causing a darkening of the film (note that both absorption spectra in Figure 1b are obtained from the same film—same thickness—and hence absorbance units directly relate to absorption coefficient of the given phase). The shift toward higher wavelengths translates into a narrowing of the bandgap. Assuming a direct bandgap for this material, we obtain a value of 1.30 eV from the absorption spectrum (see the Tauc plot in Figure S8). Contrary to Cs_2_SnI_4_ where no photoluminescence (PL) was detected, B-β CsSnI_3_ shows infrared emission with the maximum at 937 nm (1.32 eV). This implies that there is only a very small Stokes shift. Both the bandgap deduced from absorption and the maximum of the PL emission are in agreement with values reported previously.^21,22,34,35^ These properties make B-β CsSnI_3_ also interesting for alternative applications such as a light absorber for solar cells or as the emitter for near-infrared light-emitting diodes.^36,37^

**Figure 2:**
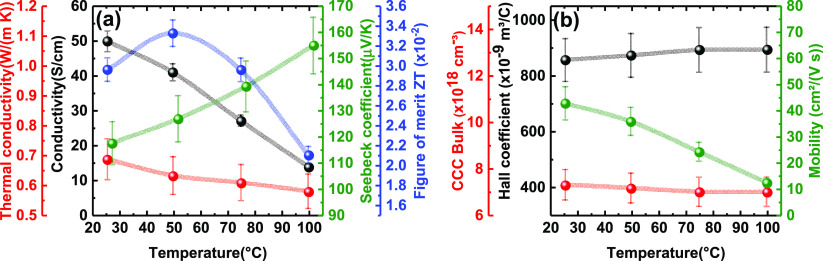
(a) Temperature dependence of electrical conductivity, σ (black line), thermal conductivity, κ (red line), Seebeck coefficient, *S* (green line), and figure of merit, ZT (blue line) of B-β CsSnI_3_. (b) Temperature dependence of Hall coefficient (black line), charge carrier concentration of the bulk, *CCC* (red line), and charge mobility (green line) of B-β CsSnI_3_. Errors bars come from measurement of film thickness and the deviation of the equipment (see the Experimental Methods). The ZT and charge mobility error bars originate from the combination of these errors.

The thermoelectric performance of B-β CsSnI_3_ thin-films was then studied under an inert atmosphere through temperature-dependent measurements ranging from 25 to 100 °C (Figure 2a) with an integrated thin film analyzer from Linseis (see the Experimental Methods).^38^ The electrical conductivity (σ) decreases with increasing temperature as expected for bandlike charge transport. A maximum conductivity of 50 ± 3 S/cm at room temperature is obtained. This is 7 orders of magnitude higher than the conductivity of the phase that was formed prior to annealing the film, Cs_2_SnI_4_. Hall effect measurements (Figure 2b) show that the B-β CsSnI_3_ thin-films are a p-type semiconductor (positive Hall coefficient) with a charge carrier concentration (*CCC*) around 7 × 10^18^ cm^–3^ and hole mobility of 42 ± 6 cm^2^ V^–1^ s^–1^ at room temperature. A temperature increase causes a sharp decrease in the charge carrier mobility in line with the observed decrease in the electrical conductivity, whereas the carrier concentration remains virtually constant. It is known that the origin of the p-type conduction of B-β CsSnI_3_ arises from its ability to accommodate Sn^4+^ ions and Sn vacancies due to oxidation, which effectively acts as self-doping and hence transit to a hole-doped state.^39^ When increasing the temperature, holes delocalize and molecular vibrations increase to achieve a metal-like behavior translating into a decrease in the electrical conductivity.^20,25,40^ Thus, the mean free path of electrons is reduced, and their mobility is also decreased. While this behavior is typical of a metal and not of an intrinsic semiconductor, we should stress that CsSnI_3_ is effectively a self-doped semiconductor (not a metal, as it clearly presents a bandgap of 1.3 eV as shown in Figure 1). In the words of Chung et al.,^20^ “although stoichiometric CsSnI_3_ is a semiconductor, the material is prone to intrinsic defects associated with Sn vacancies. This creates highly mobile holes which cause the materials to appear metallic.” We hypothesize that as no phase transition took place here upon heating and samples were stored in nitrogen during measurement, the formation of new Sn^4+^ centers is avoided, so the carrier concentration is kept constant. These findings were previously reported by others as they did not consider phase transitions upon heating B-β CsSnI_3_ to have an effect on the charge transport properties.^20^

On the other hand, the ultralow thermal conductivity (κ) detected in the whole range of temperatures is consistent with the ultralow lattice thermal conductivity reported for most halide perovskites.^14,41^ The cause of this phenomenon is ascribed to the phonon–phonon scattering inside the crystal lattice, which intensifies by increasing the temperature, hindering the thermal transport (decreasing κ).^17,24,25^ Such low thermal conductivities are beneficial for the thermoelectric performance and are found in other materials such MAPbI_3_ or SnSe, whose ZT is one of the highest reported so far.^15,42^

The positive Seebeck coefficient, *S*, as shown in Figure 2a, confirms that B-β CsSnI_3_ is a p-type semiconductor, consistent with our Hall effect measurements and in corroboration with findings from others.^20,43^*S* steadily rises up to 154 ± 11 μV/K with increasing temperature, which agrees with the decrease in electrical conductivity and is comparable to other Sn-based perovskites.^3,25^ A similar value for *S* was determined independently with a different characterization methodology (see Figure S9 and the Experimental Methods). The thermoelectric figure of merit, ZT, for our thin films (thickness = 300 nm) of B-β CsSnI_3_ ranges from 0.021 to 0.033 in the temperature range studied (room temperature to 100 °C) and reaches its maximum (0.0333 ± 0.0013) at 50 °C, which is relevant for applications near room temperature.

**Figure 3:**
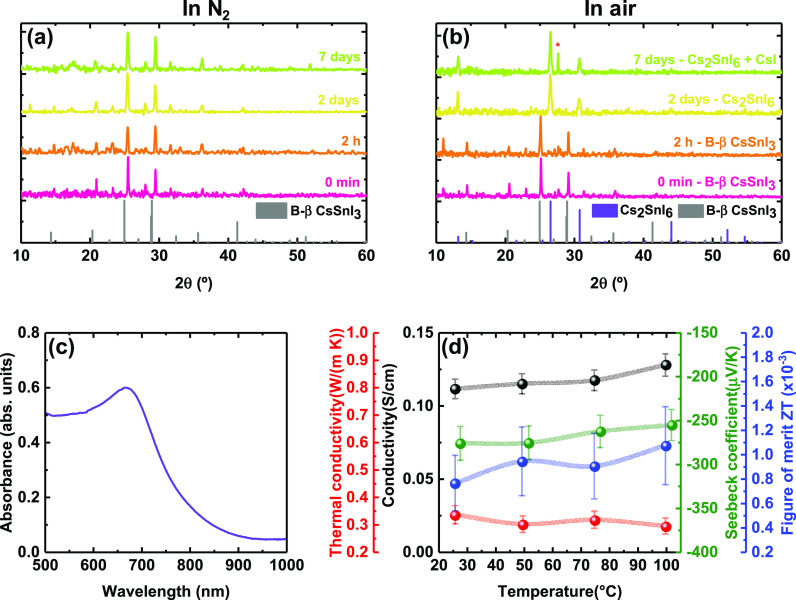
XRD diffractograms of the evolution of Cs_2_SnI_6_ thin films exposed to (a) air and (b) N_2_ for 1 week. The red asterisk stands for the main XRD peak of CsI, a byproduct of the degradation. (c) UV–vis absorbance spectrum of Cs_2_SnI_6_. (d) Temperature dependence of electrical conductivity, σ (black line), thermal conductivity, κ (red line), Seebeck coefficient, *S* (green line), and figure of merit, ZT (blue line), of Cs_2_SnI_6_ thin films. See the error bar calculation in Figure 2a.

When the B-β CsSnI_3_ thin film is kept in an inert atmosphere (Figure 3a), the black beta phase is preserved for at least 7 days. However, as mentioned, B-β CsSnI_3_ spontaneously evolves to Cs_2_SnI_6_ upon exposure to air in 2 days or less (Figure 3b). This phase change is accompanied by a decrease in the film homogeneity and the formation of pinholes spread all over the film surface, as SEM images evidence (Figure S10). The poor morphology is likely attributed to an irregular oxidation from B-β CsSnI_3_ to Cs_2_SnI_6_, which could be avoided by controlling the atmosphere composition and deposition process.^44,45^ After 1 week in air we observe the formation of some small amount of CsI, indicating some degradation of this Cs_2_SnI_6_ structure and a low stability of the phase upon exposure to air for long periods. In contrast, Figure S11 shows that the XRD diffraction pattern after heating to 150 °C in ambient conditions remains unchanged, revealing the high thermal stability of Cs_2_SnI_6_. This finding was previously confirmed by other authors who performed thermogravimetric analysis (TGA), showing no decomposition until 515 K.^46^

The conversion of B-β CsSnI_3_ to Cs_2_SnI_6_ leads to a change of the optical properties of the thin films. The shape and the intensity of the optical absorbance are completely different from the B-β CsSnI_3_ phase (Figure 3c). The absorption spectra has an overall strongly reduced absorbance compared to that of the B-β CsSnI_3_ phase.^47,48^ A direct bandgap of 1.49 eV can be estimated from the Tauc plot (Figure S12), in line with previous reports for this phase.^22,49,50^ No significant PL was detected for this material, which could be ascribed to a high nonradiative recombination rate of the films induced by the formation of trap states.^44,48,51^

Figure 3d shows the temperature dependence of the thermoelectric performance of Cs_2_SnI_6_ thin films. We observe a conductivity around 0.1 S/cm in the whole temperature range, in line with other reported values.^45^ This is 2 orders of magnitude lower than that observed previously for B-beta CsSnI_3_ (Figure 2). An ultralow thermal conductivity is also found for Cs_2_SnI_6_, together with a negative Seebeck coefficient over the whole range of temperatures, indicating that electrons are the dominant charge carriers (n-type conduction). The n-type character of the compound comes from the presence of iodine vacancies and tin interstitials inside the lattice, defects easily formed in this double perovskite.^46,51^ With our home-built setup, measurements run on a different day and on a different sample yielded a Seebeck coefficient at 25 °C of −514 μV/K (Figure S13), which is considerably higher in absolute value, although in the same order of magnitude as what was obtained from the analysis using the thin-film analyzer. We hypothesize that the difference of Seebeck values arises from a dissimilar oxidation level of the layers. It seems plausible that variations in the atmosphere, for example, oxygen concentration, moisture, or temperature, during the oxidation of B-β CsSnI_3_ to Cs_2_SnI_6_ may cause a different degree of oxidation affecting the charge transport mechanisms and, thus, the thermoelectric performance. We should also note that the oxidation in air from CsSnI_3_ to Cs_2_SnI_6_ is likely accompanied by formation of SnO_2_ which may be amorphous and hence not detected here by XRD. It is possible that this side-product also affects the overall thermoelectric properties of the film. Anyhow, the low electrical conductivity (2 orders of magnitude lower than that of B-β CsSnI_3_) leads to ZT values around 1 × 10^–3^ in the 25–100 °C range. The improvement of the electrical conductivity is thus paramount to boost the possibilities in TE of this material.

## Conclusions

In conclusion, we report the thermal deposition of Cs_2_SnI_4_ directly from the SSVD of ball-milled CsSnI_3_ powder. Absorption spectra of these thin films reveal the presence of the 2D perovskite (*E*_g_ = 1.42 eV) together with traces of Y CsSnI_3_ (*E*_g_ = 2.45 eV). Their electrical conductivity is found to be extremely low but rises almost 7 orders of magnitude after annealing for 25 min at 150 °C, when the B-β CsSnI_3_ phase is formed. B-β CsSnI_3_ shows ultralow thermal conductivity and p-type conduction corroborated from Seebeck and Hall measurements. Its electrical conductivity decreases when increasing the temperature, caused by the reduction in charge mobility. In the temperature range studied (RT–100 °C) and without exposure to air or further external doping, the maximum ZT achieved is 0.0333 ± 0.0013 at 50 °C. The black beta phase is conserved in nitrogen for more than 7 days but oxidizes into Cs_2_SnI_6_ upon exposure to air for a few hours. Cs_2_SnI_6_ has a lower electrical conductivity, an ultralow thermal conductivity, and a larger absolute Seebeck coefficient, reaching a ZT of 0.0011 ± 0.0003 (100 °C) (see Table 2 for summarized maximum values achieved). Hence, both B-β CsSnI_3_ and Cs_2_SnI_6_ show potential for implementation in low-temperature operating TEGs when the conductivity of these materials can be controllably improved.

**Table 2:** Comparison of Maximum Electrical Conductivity (σ_max_), Thermal Conductivity (κ_max_), Seebeck Coefficient (*S*_max_), and Figure of Merit ZT (ZT_max_) for the Three Species Studied in This Work^a^

phase	σ_max_ (S/cm)	κ_max_ (W/m·K)	*S*_max_ (μV/K)	ZT_max_
Cs_2_SnI_4_	2.47 × 10^–6^ at 25 °C	ND	ND	ND
B-β CsSnI_3_	50 ± 3 at 25 °C	0.69 ± 0.07 at 25 °C	154 ± 11 at 100 °C	0.0333 ± 0.0013 at 50 °C
Cs_2_SnI_6_	0.128 ± 0.007 at 100 °C	0.33 ± 0.03 at 25 °C	–255 ± 18 at 100 °C	0.0011 ± 0.0003 at 100 °C

a Temperatures at which they are achieved are given. ND stands for nondetected.

## Experimental Methods

### Materials

Cesium iodide (CsI, >99%) was purchased from TCI. Tin(II) iodide (SnI_2_, 99.999%) was purchased from Alfa Aesar. All chemicals were stored in a nitrogen-filled glovebox and used as received without further purification.

### Mechanochemical Synthesis

Stoichiometric amounts of CsI and SnI_2_ were introduced inside a 10 mL zirconia ball-mill jar with two zirconia beads of 10 mm diameter inside a nitrogen-filled glovebox. Ball-milling (BM) was performed with a MM-400 shaking ball-mill from Retsch at a frequency of 30 Hz for 30 min.

### Thin-Film Deposition by Single-Source Vacuum Deposition (SSVD)

In a typical deposition, an alumina thermal crucible (Creaphys GmbH) was placed inside a vacuum chamber, and the as-synthesized CsSnI_3_ powder was loaded. Then, the chamber was evacuated to a pressure of 7 × 10^–6^ mbar, and the source was rapidly heated to 500 °C. The deposition was stopped after the complete evaporation of the solid. The sample film thickness was measured with a mechanical profilometer (Ambios XP200). Results shown in the main text are obtained from thin films of B-β CsSnI_3_ and Cs_2_SnI_6_ with a film thickness of 210 nm (±0.5%) and 540 nm (±0.2%), respectively. The reason a thicker film was deposited to evaluate Cs_2_SnI_6_ is that the conductivity is lower than for CsSnI_3_, and therefore the overall conductance of the film falls below the limit of detection of the instrumental setup if a 210 nm film is employed. The thickness evaluation is performed on the relevant phase right before thermoelectric characterization to ensure accurate conductivity assessment. It must be noted, however, that the 540 nm thickness of the Cs_2_SnI_6_ film is obtained from a precursor B-β CsSnI_3_ film of 515 nm, meaning that an ≈5% thickness increase occurs upon phase transition.

### XRD Characterization

X-ray diffraction was measured with a powder diffractometer (Empyrean from Panalytical) equipped with a Cu Kα anode operated at 45 kV and 40 mA. Single scans were acquired in the 2θ = 8°–60° range with a step size of 2θ = 0.01° in Bragg–Brentano geometry in air. A sealed dome sample holder (Anton Paar) was used for measurements in N_2_.

### Optical Characterization

UV–vis absorption spectra of the films are acquired in a transmission configuration coupled to an Avantes Avaspec-2048L optical detector (Avantes BV). Photoluminescence measurements were performed inside a nitrogen-filled glovebox by using a MatchBox laser as an excitation source at 515 nm coupled to an Avantes Avaspec-2048L optical detector. For a typical analysis, one scan with an integration time of 3 s was collected.

### Scanning Electron Microscopy (SEM)

SEM images were obtained by using a Phenom XL G2Microscope from Thermo Fisher at an operating acceleration voltage of 10 kV. EDX data were acquired with a Hitachi S-4800 scanning electron microscope.

### Thermoelectric Property Measurements

The electrical conductivity, thermal conductivity, Seebeck coefficient, and Hall effect were measured simultaneously on the same sample by using a thin film analyzer (TFA) from Linseis. The operation mechanism of this equipment is described elsewhere.^38^ A home-built setup was employed to measure the Seebeck coefficient of B-β CsSnI_3_ and Cs_2_SnI_6_ thin films for comparison. The Seebeck coefficients of samples processed under the same conditions were measured with both setups to compare values.^52^ All measurements were performed under an inert atmosphere. For error bar calculation, we have incorporated the error of the equipment provided by the manufacturer and the thickness measurement into consideration. Final errors are ±10% for thermal conductivity, ±6% for electrical conductivity, ±7% for the Seebeck coefficient, and ±9% for the Hall coefficient. ZT and charge mobility errors are calculated from the combination of these errors.

## Supporting Information Available

XRD diffractograms of as-synthesized CsSnI_3_; fitted XRD diffractogram, Tauc plot and *J*–*V* curve of Cs_2_SnI_4_ thin films; XRD diffractograms of different batches and annealing effect on Cs_2_SnI_4_ and conversion to B-β CsSnI_3_; Tauc plot and Seebeck coefficient calculation of B-β CsSnI_3_ thin films; XRD diffractograms of annealing effect on Cs_2_SnI_6_; Tauc plot and Seebeck coefficient calculation of Cs_2_SnI_6_ thin films; SEM images of Cs_2_SnI_4_, B-β CsSnI_3_, and Cs_2_SnI_6_ thin films (PDF)
