# Supplementary material for: Vacuum-Deposited Cesium Tin Iodide Thin Films with Tunable Thermoelectric Properties
Source: ACS Appl Energy Mater. 2022 Jul 26;5(8):10216–23. doi: 10.1021/acsaem.2c01936 (PMC9400028; doi:10.1021/acsaem.2c01936)
Supplement: Supplementary file 1 — ae2c01936_si_001.pdf [file ae2c01936_si_001.pdf]

Supporting Information:

Vacuum Deposited Cesium Tin Iodide Thin Films with Tunable Thermoelectric Properties

*Paz Sebastia-Luna,[a] Unnati Pokharel,[b] Bas A. H. Huisman,[a] L. Jan Anton Koster,[b] Francisco Palazon,[*a,c] and Henk J. Bolink,[*a]*

[a] Instituto de Ciencia Molecular, ICMol, Universidad de Valencia, 46980 Paterna, Spain

[b] Zernike Institute for Advanced Materials, University of Groningen, 9747 AG Groningen, The Netherlands

[c] Departamento de Ingeniería Química y Ambiental, Universidad Politécnica de Cartagena, 30202 Cartagena, Spain

Corresponding Authors

*francisco.palazon@uv.es

*henk.bolink@uv.es

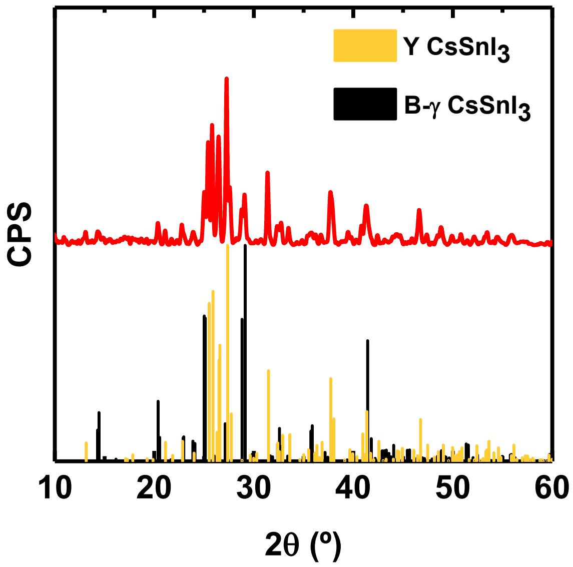

Figure S1. XRD diffractogram of as-synthesized $CsSnI_3$ powder by dry mechanochemistry together with the Inorganic Crystal Structure Database patterns of yellow phase (Y $CsSnI_3$, ICSD code 262927) and the black gamma phase (B-γ $CsSnI_3$, ICSD code 262926).

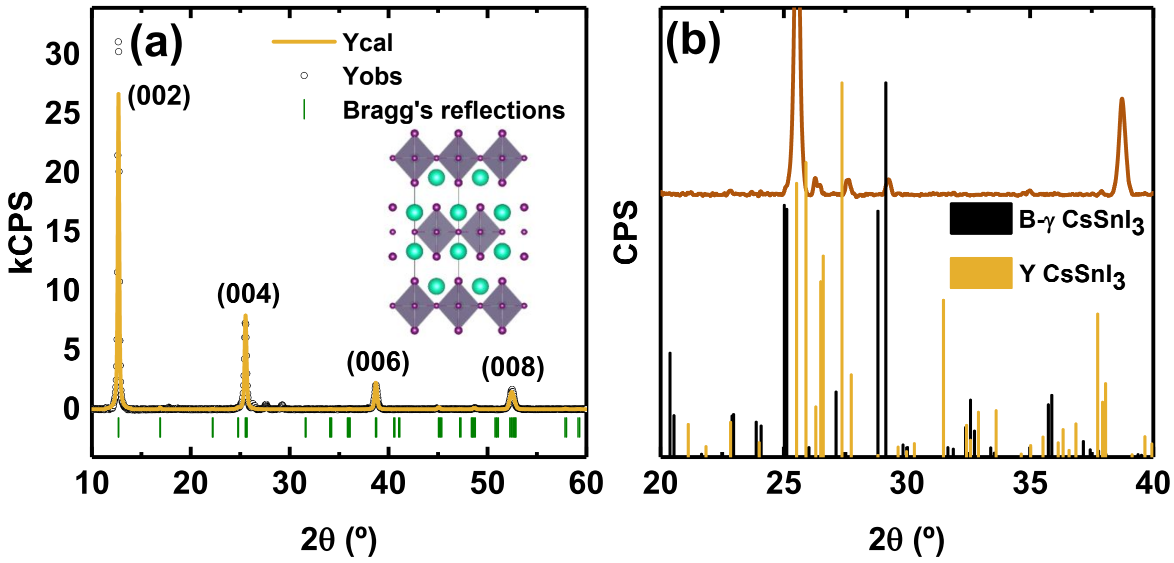

Figure S2. (a) Experimental (open circles) and fitted (yellow line) diffractograms of as-deposited Cs_2SnI_4 thin-films showing planes with preferential orientation (bold). Calculated Bragg's reflections position in green. Inset with the expected crystallographic structure of Cs_2SnI_4 is given. (b) Zoomed area of as-deposited Cs_2SnI_4 thin-film together with the Inorganic Crystal Structure Database patterns of yellow phase (Y $CsSnI_3$, ICSD code 262927) and the black gamma phase (B-γ $CsSnI_3$, ICSD code 262926).

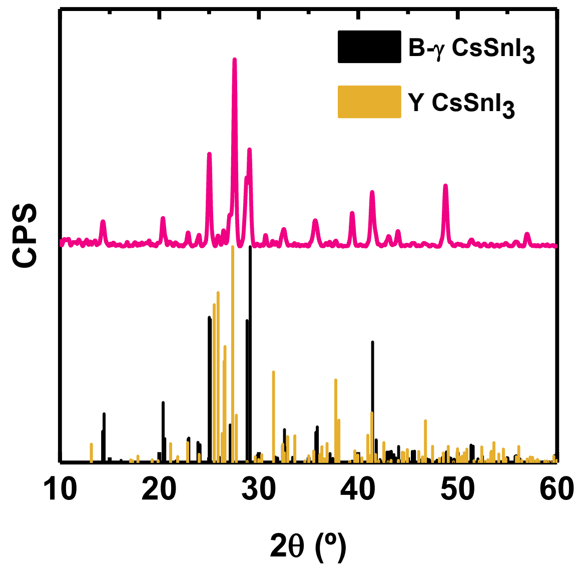

Figure S3. XRD diffractogram of as-synthesized Cs_2SnI_4 powder by dry mechanochemistry. As-synthesized powder is a mixture of yellow phase (Y $CsSnI_3$, ICSD code 262927) and the black gamma phase (B-γ $CsSnI_3$, ICSD code 262926), as clearly indicated in the data.

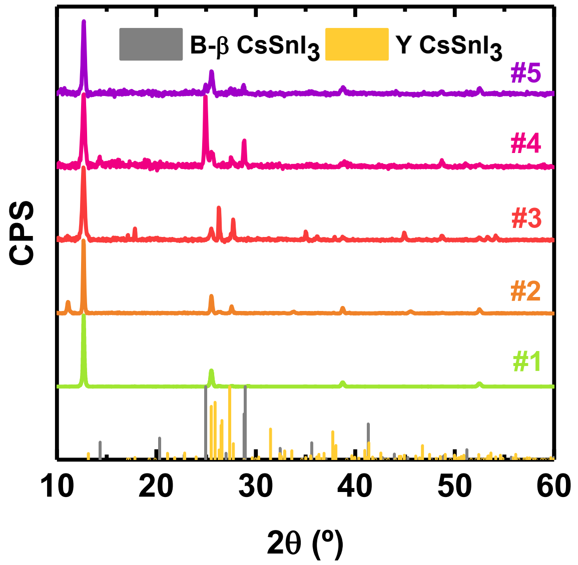

Figure S4. XRD diffractogram of as-deposited Cs_2SnI_4 thin-films from different deposition batches together with the Inorganic Crystal Structure Database patterns of yellow phase (Y $CsSnI_3$, ICSD code 262927) and the black beta phase (B-β $CsSnI_3$, ICSD code 262925).

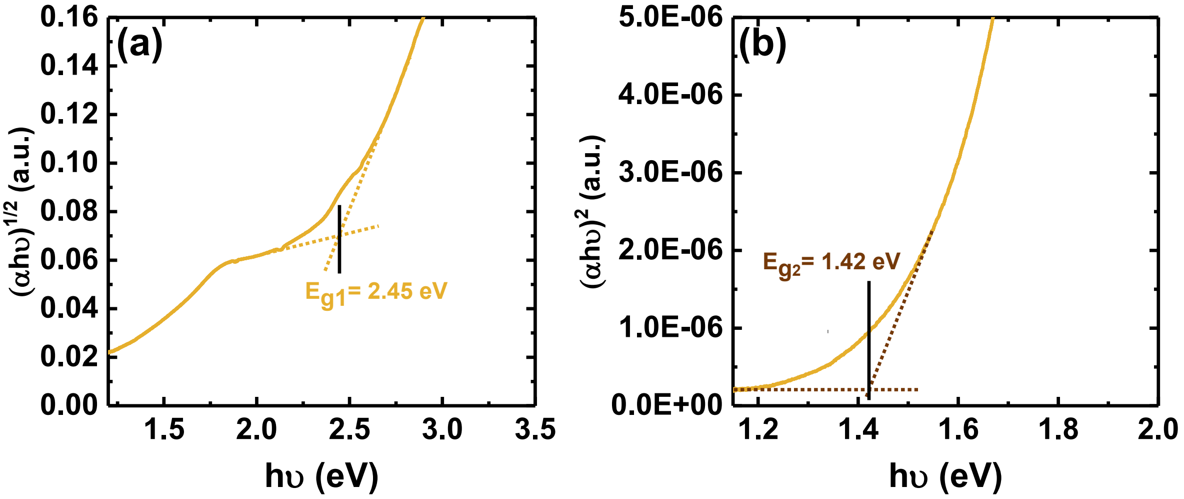

Figure S5. Tauc plot of as-deposited SSVD $CsSnI_3$ thin films considering (a) an indirect and (b) direct transition. E_{g1} is ascribed to the yellow phase of $CsSnI_3$ and E_{g2}, to Cs_2SnI_4.

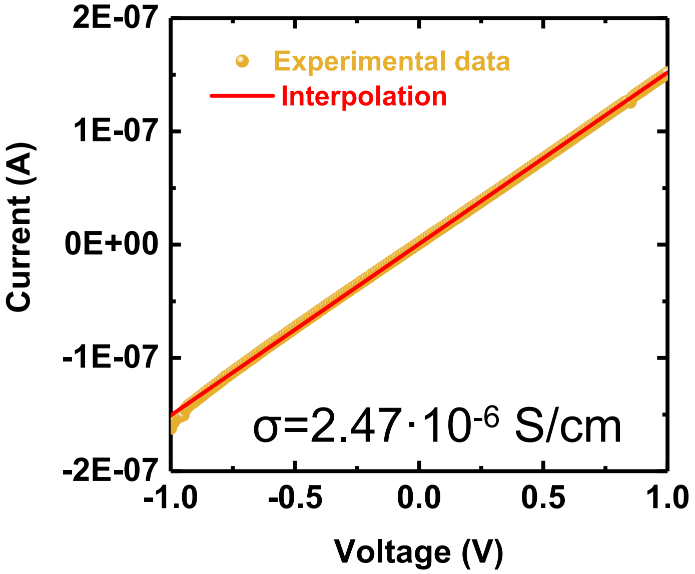

Figure S6. Current vs voltage curve of Cs_2SnI_4 (yellow line) to determine the electrical conductivity. Red line represents the linear interpolation of the JV curve. According to Ohm's law (Eq. S1), it is possible to calculate the electrical conductivity from the slope of the linear plot.

$$I = \frac{1}{R}V = \sigma V \qquad \textit{Eq. S1}$$

Where I stands for current intensity, R electrical resistance, V voltage and σ electrical conductivity.

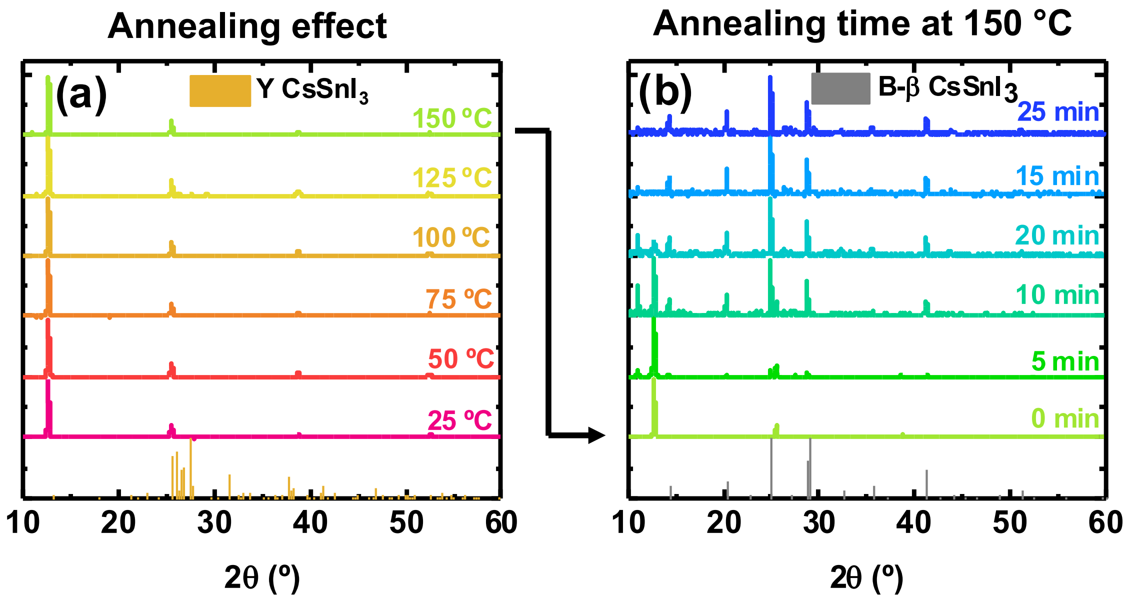

Figure S7. XRD diffractograms of (a) in-situ annealing of Cs_2SnI_4 thin films up to 150 ºC and (b) effect of the annealing time at 150 ºC until it is fully converted into the black beta phase of $CsSnI_3$.

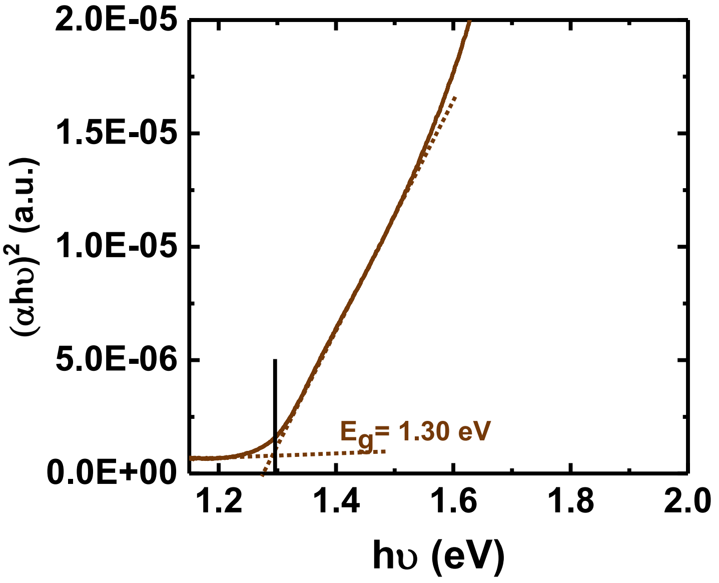

Figure S8. Tauc plot of B-β $CsSnI_3$ thin films considering a direct transition. A bandgap of 1.30 eV is estimated.

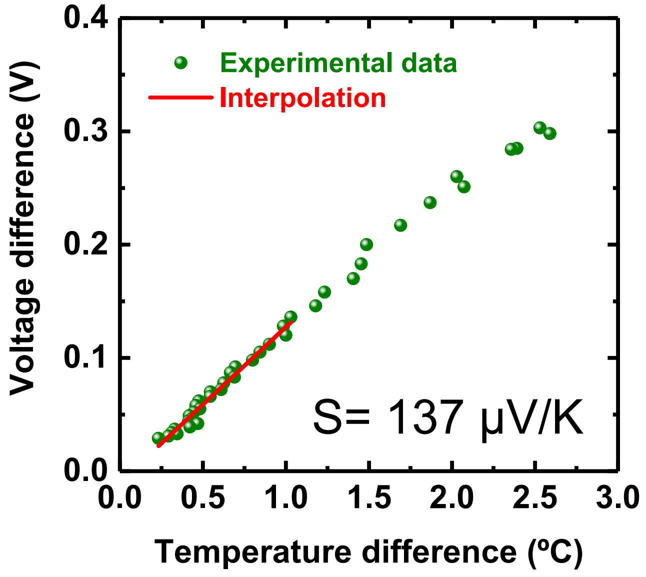

Figure S9. Thermoelectric voltage vs. temperature difference for B-β $CsSnI_3$ thin films at room temperature. The sign and the value of the Seebeck coefficient are given by Eq. S2.

$$S = -\frac{\Delta V}{\Delta T} \qquad \textit{Eq. S2}$$

Where ΔV stands for difference in thermal voltage and ΔV, temperature difference.

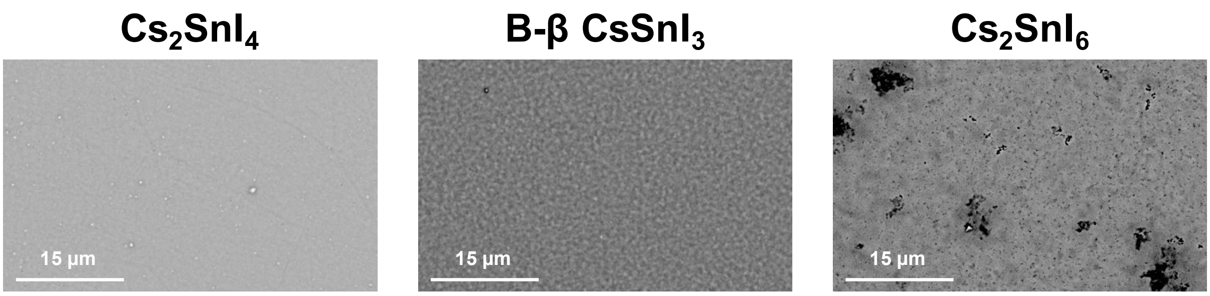

Figure S10. SEM images of Cs_2SnI_4, B-β $CsSnI_3$ and Cs_2SnI_6 thin films at 10,000x magnification.

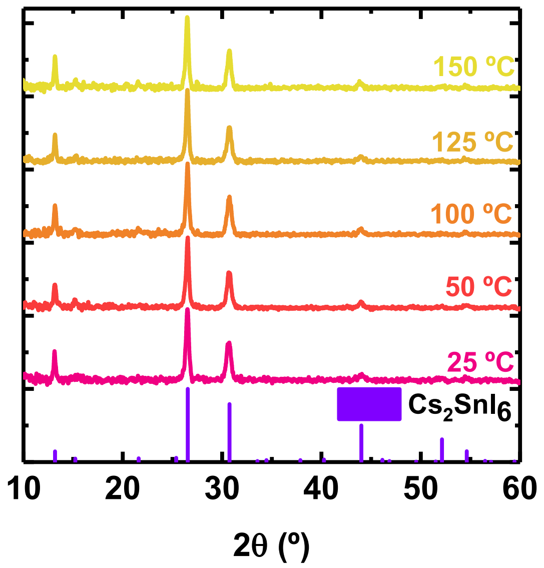

Figure S11. XRD diffractograms of in-situ annealed Cs_2SnI_6 thin films up to 150 ºC.

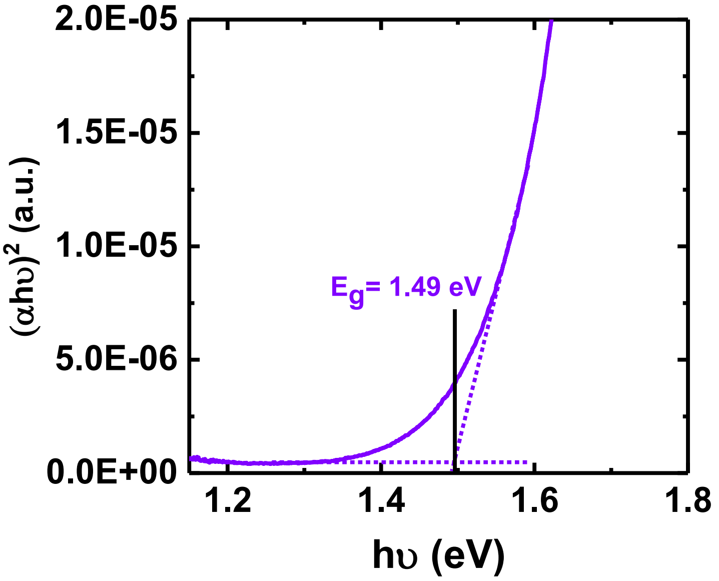

Figure S12. Tauc plot of Cs_2SnI_6 thin films considering a direct transition. A bandgap of 1.49 eV is estimated.

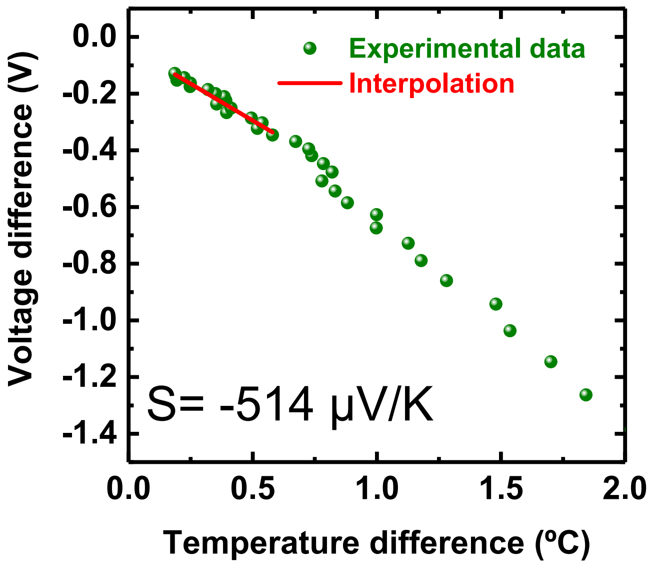

Figure S13. Thermoelectric voltage vs. temperature difference for Cs_2SnI_6 at room temperature. The sign and the value of the Seebeck coefficient are given by Eq. S2.
